# Changes of the eSheath Outer Dimensions Used for Transfemoral Transcatheter Aortic Valve Replacement

**DOI:** 10.1155/2015/572681

**Published:** 2015-04-23

**Authors:** Till Koehler, Michael Buege, Heinrich Schleiting, Melchior Seyfarth, Klaus Tiroch, Marc Vorpahl

**Affiliations:** Department of Cardiology, Zentrum für Forschung in der Klinischen Medizin (ZFKM), HELIOS Klinikum Wuppertal, Herzzentrum, University Witten/Herdecke, Arrenberger Straße 20, 42117 Wuppertal, Germany

## Abstract

Innovative catheter systems with lower-profile sheaths and a dynamic expansion mechanism (DEM) were recently introduced for transcatheter aortic valve replacement (TAVR). However, the labeling of 14 F and 16 F eSheaths denote the inner nominal diameter. Exact changes of the clinically relevant outer diameters during usage are not available. eSheaths were measured every 30 mm using a digital caliper. Unused 14 F and 16 F eSheaths served as controls. Maximum eSheath diameters were measured after insertion of the Edwards Commander Delivery System (ECDS) into 14 F and 16 F eSheaths.Finally, eSheaths were retrieved and measured after TAVR. Outer diameters of control 14 F eSheaths were 5.8 mm and 6.50 mm for the 16 F eSheath. Introduction of the 23 mm and 26 mm ECDS into 14 F eSheaths showed a maximum diameter of 7.65 mm and 7.64 mm (*P* = NS). Introduction of the 29 mm ECDS into the 16 F eSheath showed the greatest diameter of 8.18 mm (*P* = 0.03). After TAVR, diameters of the 14 F eSheaths were 7.14 mm (23 mm valve) and 7.26 mm (26 mm valve) (*P* = NS), while 16 F eSheaths were 8.10 mm (29 mm valve) (*P* ≤ 0.03). Nominal 14 F and 16 F eSheaths showed a significant increase of the outer diameter during advancement of the ECDS and after TAVR implantation.

## 1. Introduction

Vascular complications contribute significantly to the morbidity and mortality during and after transcatheter aortic valve replacement (TAVR) via femoral arterial access in randomized trials and daily practice [1–7]. Catheter systems are continuously optimized including lower-profile sheath designs [2, 3]. The current generation eSheath for the Edwards SAPIEN 3 TAVR is a nominal 14 F (~4.7 mm) or 16 F (~5.3 mm) femoral sheath with a dynamic expansion mechanism (DEM). This sheet system expands transiently during the passage of the SAPIEN 3 transcatheter heart valve and abates to a lower-profile diameter thereafter. The DEM facilitates the placement of the large femoral sheath in the frail TAVR population with atheroma-altered iliacofemoral vessels, vascular calcification burden, and vessel tortuosity and may therefore reduce adverse events at the vascular access site.

The manufacturer recommends a minimal vessel size of 5.5 mm for the 14 F eSheath (23 mm and 26 mm Sapien 3 valve) and 6.0 mm for the 16 F eSheath (29 mm Sapien 3 valve). However, the actual changes of the eSheath dimensions during the Sapien 3 transfemoral TAVR procedure are not published. Therefore, we performed an assessment of the outer diameter changes of the eSheath system before usage, during passage of the pusher through the ECDS system* ex vivo* and after the removal of the* in vivo* used eSheath. These measurements of the outer luminal diameters may help the TAVR operators to better understand the effective external dimensions of the current eSheath system during implantation.

## 2. Methods

### 2.1. eSheath Collection

**Figure 1:**
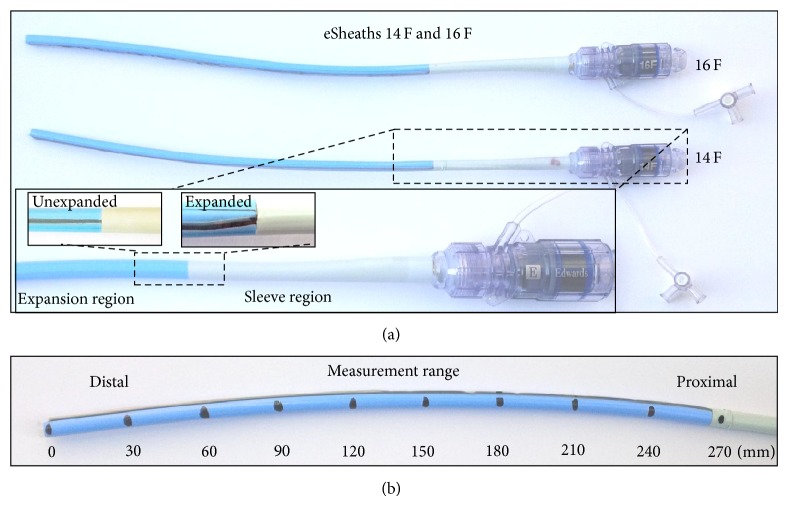
14 F and 16 F femoral eSheath for Edwards Sapien 3 TAVR. (a) The eSheath system expands transiently during the passage of the Sapien 3 transcatheter heart valve and abates to a lower-profile diameter thereafter. A circumferential plastic sleeve stabilized the expandable part of the eSheath at the intersection between the expandable part and the stiff bleed back prevention valve. (b) The outer diameter of the expandable part of the eSheath was measured by a digital caliper from the most distal part (0 mm) in 30 mm increments until the sleeve region (300 mm) was reached.

First, 14 F eSheaths (*n* = 5) and 16 F eSheaths (*n* = 5) were analyzed before usage as controls; Edwards Lifescience Irvine CA, USA (Figure 1(a)).

Second, unexpanded control eSheaths were used for further* ex vivo* measurements including advancement of the ECDS.

Third, a total of 15 eSheaths were flushed with buffered saline and analyzed before disposal after femoral Sapien 3 transcatheter valve implantation. Five 14 F eSheaths were analyzed after implantation of 23 mm Sapien 3 valves, five 14 F eSheaths were analyzed after implantation of 26 mm Sapien 3 valves, and five 16 F eSheaths were analyzed after implantation of 29 mm Sapien 3 valves.

The study was approved by the local ethics committee and all patients provided written informed consent.

### 2.2. Digital Caliper

Measurements were performed using an ISO certified iGaging ABSOLUTE ORIGIN metric digital electronic caliper meeting DIN Standard 862 (resolution: 0.0005^″^/0.01 mm; accuracy: 0.001^″^/0.02 mm; range: 6^″^/150 mm), San Clemente, CA, USA. The caliper was calibrated to zero before each measurement.

### 2.3. eSheath Diameter Analyses

**Figure 2:**
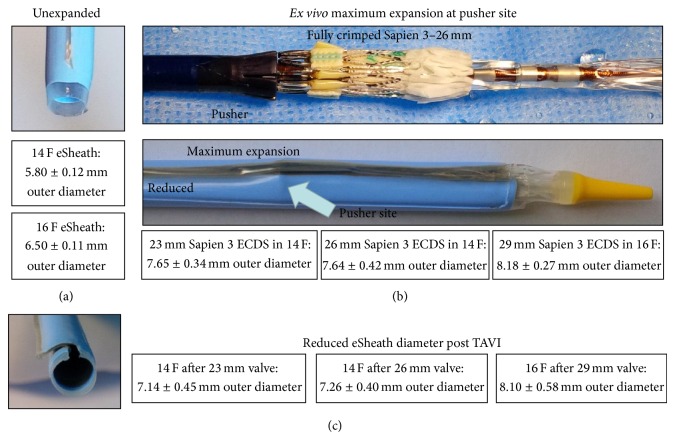
Outer diameter dimensions of the eSheath. (a) Outer diameter of the unexpanded 14 F eSheath was 5.80 ± 0.12 mm and 6.50 ± 0.11 mm for the 16 F eSheath. (b)* Ex vivo* maximum expansion diameter at the pusher site during the passage of the fully crimped 26 mm Sapien 3 valve. Maximum expansion diameter was 7.65 ± 0.34 mm with a 23 mm Sapien ECDS in a 14 F eSheath, 7.64 ± 0.42 mm in a 26 mm Sapien ECDS in a 14 F eSheath, and 8.18 ± 0.27 mm in a 29 mm Sapien 3 ECDS in a 16 F eSheath. (c) Expanded distal end of the 14 F femoral eSheath after TAVR without the introducer. Outer eSheath diameter was 7.14 ± 0.45 mm after implantation of a 23 mm valve in a 14 F eSheath, 7.26 ± 0.40 mm after implantation of 26 mm valve in a 14 F eSheath, and 8.10 ± 0.58 mm after implantation of a 29 mm valve in a 16 F eSheath.

**Figure 3:**
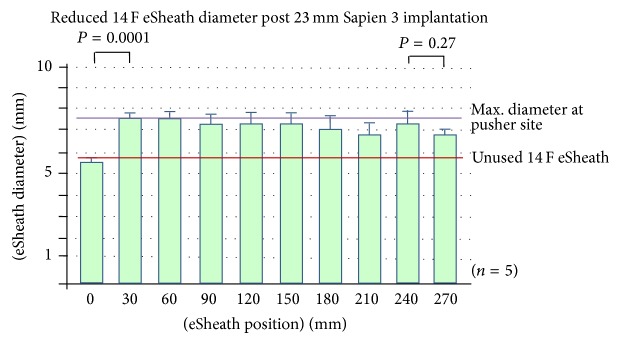
Longitudinal 14 F eSheath outer diameters at the 0, 30, 60, 90, 120 150, 180, 210, 240, and 270 mm measuring points following 23 mm Sapien 3 implantation.

The first assessment focused on the unused 14 F and 16 F eSheaths, defined as controls (*n* = 5 for each group) (Figure 2(a)). We measured the outer diameter of the expandable part of the eSheath by a digital caliper. The most distal part (0 mm) is significantly smaller in diameter which is related to the absence of the plication of the dynamic expansion mechanism at the very distal part of the eSheath (Figures 2(a), 2(c), 3, and 5). After the insertion of the appropriate introducer, we started measuring at 30 mm at the distal end with 30 mm increments until the sleeve region (300 mm) was reached (Figure 1(b)).

The second assessment aimed to determine maximum* ex vivo* changes induced by the pusher of the ECDS (Figure 2(b)). The pusher secures the position of the valve within the system during the passage through the sheath, and the diameter of the pusher is larger than the diameter of the crimped valve (Figure 2(b)). The respective ECDS for the 23 mm and 26 mm Sapien valves were used in the 14 F eSheath and the 29 mm valve ECDS in the 16 F eSheath. The balloon part of the ECDS was therefore locked outside the ECDS pusher part (*n* = 5 for each group).

Finally, we assessed the maximum outer dimensions of the eSheath following the* in vivo* TAVR implantation (*n* = 5 for each group).

### 2.4. Statistical Analysis

Results are expressed as mean ± standard deviation. The significance of variability among the means of the experimental groups was determined by 1- or 2-way ANOVA. All statistical tests were performed by using the software JMP (Version 7, SAS Institute Inc., Cary, NC, 1989–2007). Differences among experimental groups were considered statistically significant at *P* < 0.05.

## 3. Results

The Edwards eSheath represents an ultralow delivery profile. The arterial sheath consists of a flexible part (360 mm) which is placed into the femoral artery (~270 mm) except for the outer sleeve at the very proximal part (~90 mm). A rigid proximal extracorporeal section includes the bleed back prevention mechanism of the eSheath (Figure 1(a)).

### 3.1. Assessment of Unused eSheaths

The outer diameters of the flexible part of the unused 14 F and 16 F eSheaths were measured at 5.80 ± 0.12 mm and 6.50 ± 0.11 mm, respectively. Interestingly, these diameters of the unused control sheaths are already higher than the minimum arterial diameters of 5.5 mm for the 14 F and 6.0 mm for the 16 F eSheath recommended by the manufacturer (Figure 1(b)).

### 3.2. *Ex Vivo* Assessment of the Maximum eSheath Expansion at the Pusher Site

Maximum enlargement of the eSheath was measured at the pusher site of the ECDS. Therefore, we locked the balloon outside the ECDS pusher and inserted the appropriate 23 mm and 26 mm ECDS in the 14 F eSheath and the 29 mm ECDS into the 16 F eSheath. The pusher was placed into the appropriate eSheath at 60 mm from the distal expandable part. The maximum outer diameter was measured at the pusher site using a digital caliper (Figure 1).

The outer diameters of the eSheaths increased significantly after insertion of the ECDS pusher into the eSheath. Introduction of the 23 mm ECDS (7.0 mm pusher diameter) into the nominal 14 F eSheath led to a maximum eSheath dimension at the pusher site of 7.65 mm, nominally corresponding to ~23 F. The 26 mm Edwards Commander Delivery System (7.0 mm pusher diameter) showed a comparable maximum eSheath dimension at the pusher site of 7.64 mm, approximately 23 F. Finally, the advancement of the 29 mm Edwards Commander Delivery System (8.0 mm pusher diameter) into the 16 F eSheath led to a maximum eSheath dimension at the pusher site of 8.18 mm, approximately 24.5 F.

### 3.3. Assessment of the eSheath Dimensions after Sapien 3 Femoral Transcatheter Valve Implantation

**Figure 4 fig4:**
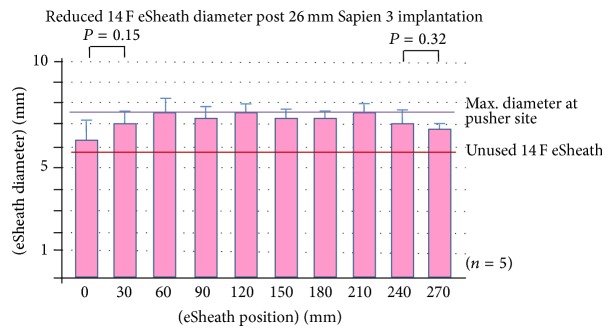
Longitudinal 14 F eSheath outer diameters at the 0, 30, 60, 90, 120 150, 180, 210, 240, and 270 mm measuring points following 26 mm Sapien 3 implantation.

**Figure 5:**
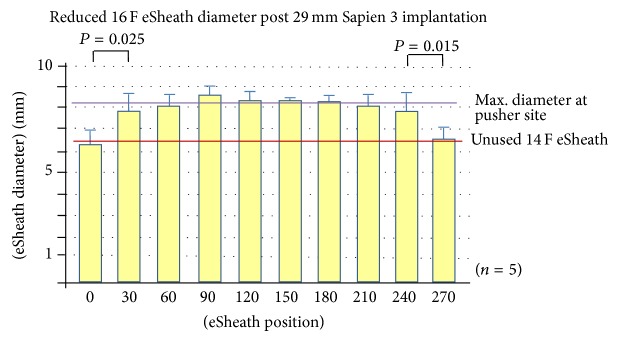
Longitudinal 16 F eSheath outer diameters at the 0, 30, 60, 90, 120 150, 180, 210, 240, and 270 mm measuring points following 29 mm Sapien 3 implantation.

We collected five 14 F eSheaths after 23 mm Sapien 3 implantation, five 14 F eSheaths after 26 mm Sapien 3 implantation, and five 16 F eSheaths after 29 mm Sapien 3 implantation. Multislice computed tomography (MSCT) of the iliac and femoral artery revealed mean minimal luminal diameter (MLD) of 7.64 ± 0.75 mm in the Sapien 23 mm group, 7.70 ± 0.87 mm in the Sapien 26 mm group (both 14 F eSheaths), and 8.48 ± 0.60 mm in the Sapien 29 mm group using 16 F eSheaths (*P* = NS for all comparisons). TAVI implantation was successful in all cases, without relevant femoral access site adverse events. The outer diameters of the eSheaths increased significantly after Sapien 3 implantation compared to the diameters mentioned above for the control sheaths. The mean outer diameter of the 14 F eSheath after 23 mm valve implantation was 7.14 ± 0.45 mm and that of the 14 F eSheath after placing the 26 mm valve was 7.26 ± 0.40 mm and it was markedly larger with 8.10 ± 0.58 mm for the 16 F eSheath after 29 mm valve implantation (*P* < 0.02 for all used eSheaths compared to unused eSheaths) (Figures 3 [Fig fig4]–5).

**Figure 6:**
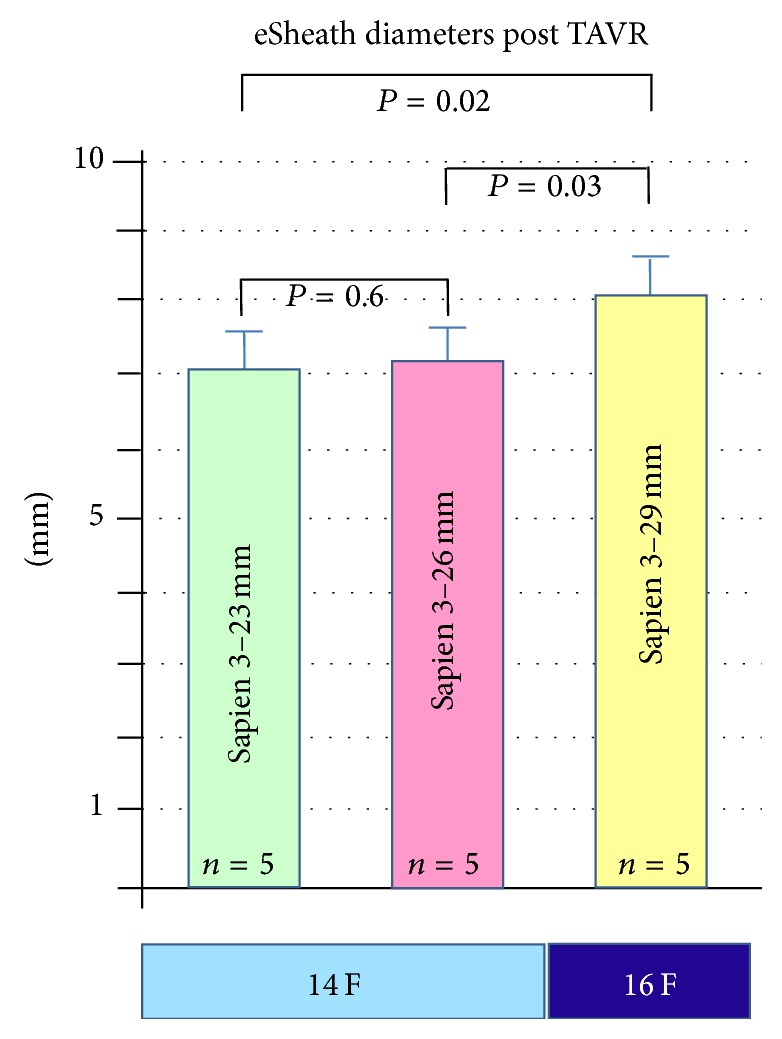
Mean outer diameters of the 14 F eSheath after Sapien 23 mm and 26 mm implantation, respectively, and of the 16 F eSheath after Sapien 29 mm implantation.

Interestingly, the 14 F eSheath showed a trend to larger outer diameters after using the 26 mm valve compared to the 23 mm valve. However, this was not statistically significant (*P* = 0.6). The 16 F eSheath showed a significant larger outer diameter after 29 mm valve implantation compared to the 14 F eSheath (*P* = 0.03) (Figure 6).

The circumferential plastic sleeve stabilized the expandable part of the eSheath at the intersection between the expandable part and the stiff bleed back prevention valve (Figures 1(a) and 5). The outer diameter of the expandable part without the sleeve (0–240 mm) of the 16 F eSheath after 29 mm valve implantation was significantly smaller than the adjacent sleeve area (270 mm) (Figures 3–5). This trend was also noted in the 14 F eSheath after 23 mm and 26 mm valve implantation, however, without reaching statistical significance.

The area of the expandable part of the 16 F eSheath after 29 mm valve implantation was significantly smaller than the adjacent 24 cm measurement at the expandable part without the sleeve (Figures 3–5). This trend was also noted in the 14 F eSheath after 23 mm and 26 mm valve implantation, however, without reaching statistical significance. In summary, the eSheath outer diameters are about 1.2 mm larger compared to the nominal inner diameter and increase by another 1.7 mm during valve passage (Table 1). Following real-world valve implantation, the observed eSheath retraction after removal is 0.1–0.5 mm.

## 4. Discussion

Transcatheter aortic valve replacement (TAVR) represents a valid option for the treatment of severe symptomatic aortic stenosis in patients at very high or prohibitive surgical risk [7]. However, vascular complications still represent a challenging limitation in transfemoral transcatheter aortic valve replacement due to significant morbidity and mortality [4–7]. These may include major and minor VARC complications, for example, aortic dissection, access site bleeding, femoral dissection, and vessel occlusion [8–11].

The introduction of lower-profile eSheaths with a dynamic expansion mechanism leads to a significant reduction of vascular complications after transfemoral TAVR [2]. This improvement enabled TAVR in a wider population including patients with diseased peripheral arteries.

The eSheath is distributed with a nominal inner lumen diameter of 14 F for the application of the 23 mm and 26 mm Sapien 3 valves and 16 F for the application of the 29 mm Sapien 3 valve. With a dynamic expansion mechanism, this sheath system adapts transiently during the passage of the Edwards Sapien 3 transcatheter heart valve and abates to a lower-profile diameter thereafter.

eSheath dimensions are suggested to be about 16–18 F for TAVR implantation, but the outer diameters are about 1.2 mm larger. These increase by another 1.7 mm during valve passage. However, significantly larger dimensions are reached at the site of the pusher during implantation, although this is limited to a short period of time during valve passage.

In addition, the eSheath does not retract fully to the native dimensions after valve passage, as seen after complete assessment of the used eSheaths. The retraction of mean eSheath outer diameter after removal observed in our study was on average 0.5 mm for the 14 F eSheath and only 0.1 mm for the 16 F eSheath. This suggests that the expansion following passage of the large 29 mm valve is less reversible compared to the smaller valves (Figure 6).

The introduction of lower-profile sheaths has undoubtedly improved vascular complications rates as a major limitation of the TAVR procedure. However, we believe that the TAVR community may have a particular interest in knowing the effective outer eSheath diameters during valve advancement to further improve patient outcome.

*Limitations*. We did not measure the loaded Sapien 3 valve within the eSheath* ex vivo* to avoid any contamination or damage of the valve/system prior to implantation.

## 5. Conclusion

Nominal 14 F and 16 F luminal eSheath systems showed significant outer diameter changes during and after advancement of the appropriate Edwards Commander Delivery System. This information may be of significant value for patient selection suggested for TAVR.

## Figures and Tables

**Table 1:** Summary of unexpanded, maximum, and mean eSheath outer diameters of 14 F and 16 F eSheaths.

eSheath outer diameter (mm)	14 F eSheath/23 mm Sapien	14 F eSheath/26 mm Sapien	16 F eSheath/29 mm Sapien	*P* value (23 versus 29 mm)
Unexpanded eSheath	5.80 ± 0.12 mm	5.80 ± 0.12 mm	6.50 ± 0.11 mm	0.0001
*eSheath ex vivo* max. at pusher site	7.65 ± 0.34 mm	7.64 ± 0.42 mm	8.18 ± 0.27 mm	0.03
Mean eSheath diameter after TAVI	7.14 ± 0.45 mm	7.26 ± 0.40 mm	8.10 ± 0.58 mm	0.02

## Abbreviations

DEM: Dynamic expansion mechanism
ECDS: Edwards Commander Delivery System
F: French
LPS: Low-profile sheath
TAVR: Transcatheter aortic valve replacement
TAVI: Transcatheter aortic valve implantation
MSCT: Multislice computed tomography
MLD: Minimal luminal diameter.
